# Quality of life and correlation with clinical and radiographic variables in patients with ankylosing spondylitis: a retrospective case series study

**DOI:** 10.1186/s12891-017-1711-1

**Published:** 2017-08-15

**Authors:** Ji-chen Huang, Bang-ping Qian, Yong Qiu, Bin Wang, Yang Yu, Ze-Zhang Zhu, Jun Hu, Zhe Qu

**Affiliations:** 1 Department of Spine Surgery, Drum Tower Hospital, the Affiliated Hospital of Nanjing University Medical School, Zhongshan Road 321, Nanjing, 210008 China; 2 Medical School of Southeast University, Nanjing, China

**Keywords:** Ankylosing spondylitis, Clinical variables, Radiographic variables, Quality of life, Oswestry disability index, Physical function

## Abstract

**Background:**

Previously, many studies have evaluated quality of life (QoL) in patients with ankylosing spondylitis (AS), however, none of them specifically investigated the correlation between pain-related disability measured by Oswestry Disability Index (ODI) and QoL in AS patients. In addition, the correlation between global kyphosis (GK) in lateral plain radiographs and QoL in AS patients remains unclear up to now. Therefore, this study aimed to evaluate QoL and correlation with clinical and radiographic variables in AS patients, especially to figure out the relationship about the pain-specific disability measured by ODI, GK and QoL.

**Methods:**

From January 2008 to November 2015, two hundred and forty-five consecutive patients with an average age of 36.2 ± 10.9 years (range, 17–66 years) satisfying the Modified New York Criteria for AS from a single institution were enrolled. Bath Ankylosing Spondylitis Disease Activity Index (BASDAI), Bath Ankylosing Spondylitis Functional Index (BASFI), Bath Ankylosing Spondylitis Metrology Index (BASMI) and Bath Ankylosing Spondylitis Global score (BAS-G) were applied to assess the disease activity, functional status, spinal mobility and overall feeling of AS patients, respectively. ODI was recorded to evaluate low back pain-related disability. QoL was evaluated by the Short Form-36 (SF-36). According to global kyphosis (GK) measured on standing lateral full-spine radiographs, the patients were divided into two groups: mild kyphotic group (GK < 70°,*n* = 176) and severe kyphotic group (GK ≥ 70°,*n* = 69).

**Results:**

The scores of BASDAI, BASFI, BASMI and ODI had significant negative correlations with all SF-36 subscale scores (*P* < 0.01). BASFI and BASMI scores of severe kyphotic group were much higher than those of mild kyphotic group, respectively (*P* = 0.005 and *P* = 0.001, respectively) and the score of physical function (PF) subscale in severe kyphotic group was significantly higher than that in mild kyphotic group (*P* = 0.046) as well. Notably, the scores of ODI, BASFI and BASMI were the major predictors of PF subscale score of SF-36.

**Conclusions:**

Poor QoL is significantly correlated with high disease activity, poor functional status and decreased spinal mobility in AS. GK is significantly associated with functional status, spinal mobility and QoL in AS patients. ODI, BASFI and BASMI are the major predictors of PF subscale of SF-36.

## Background

Ankylosing spondylitis (AS) is a chronic progressive inflammatory disorder that predominantly involves the axial skeleton, possibly causing sacroiliitis, spondylitis, spondylodiscitis, enthesitis and arthritis of the zygoapophyseal, costovertebral and costosternal joints [1]. In the late stage of AS, thoracolumbar kyphosis often occurs.

Pain, stiffness and fatigue are cardinal complaints in AS patients [2, 3]. These clinical symptoms and subsequent disease progression result in substantial functional limitations, gradual loss of spinal mobility and impairment of quality of life (QoL). Health-related QoL measurement objectively reflects the actual effect of the disorder on an individual and the degree of suffering [4, 5]. Knowing which aspects of QoL are most affected by a particular disease is helpful for both researchers and clinicians to identify the disease-related problems that may be inadvertently neglected in the research or clinical practice [6]. Also, the medical outcomes survey short form-36 (SF-36) has been frequently and widely used for rheumatologic and musculoskeletal disorders [7–10]. In previous studies, SF-36 is the most commonly used measurement technique to assess the health-related QoL of AS patients [10–18]. Among these studies, the relationship between QoL and disease activity or functional status has been investigated by many researchers [13–16]. However, relatively fewer studies focused on the relationship between spinal mobility and QoL measured by SF-36 in AS [13, 17, 18].

First reported in 1980, the Oswestry Disability Index (ODI) is a validated and well- accepted measure of the impact of back pain or leg pain on disability [19]. Moreover, ODI has been widely used for the clinical evaluation of AS patients [20–22]. However, the relationship between ODI and QoL in AS patients remains unknown so far.

To our best knowledge, a limited number of cases were enrolled in most of the previous studies which focused on the QoL in AS patients. Furthermore, no studies have specifically investigated the relationship between the disability measured by ODI and QoL in AS patients. In addition, the correlation between global kyphosis (GK) in lateral plain radiographs and QoL in AS patients still remains unclear.

The aim of the study was to evaluate QoL and correlation with clinical and radiographic variables in AS patients, especially to figure out the relationship between the pain-related disability measured by ODI, GK and QoL.

## Methods

### Study design

The study was conducted in compliance with the Helsinki Declaration to protect human subjects and was approved by the Medical Ethics Committee of Medical School of Nanjing University (the ethics approval number provided by the board was 2,011,052). Written informed consent was obtained from all patients prior to testing. Study participants were recruited from outpatients and inpatients of spine surgery department of one tertiary referral hospital in China. Between January 2008 and November 2015, 245 consecutive patients who fulfilled the modified New York criteria for AS were included [23]. Among these patients, 120 patients received drug therapy in the clinic and 125 patients were admitted for spinal surgery for correction of thoracolumbar kyphosis. Patients with concomitant disorders such as serious infections or chronic diseases (cardiac, respiratory, gastro-intestinal, neurological, endocrine, etc) were excluded. Patients with spinal surgery history were also excluded to eliminate the effect of prior spinal surgery on clinical variables and QoL.

The information of patients were collected by two trained orthopaedic residents using identical questionnaires including demographic information (age, gender, age at onset, disease duration), disease-specific characteristics (overall pain, fatigue, degree and duration of morning stiffness, low back pain-related disability, peripheral arthritis, nonsteroidal antiinflammatory drugs (NSAIDs) and anti-tumor necrosis factor (TNF) drugs intake, erythrocyte sedimentation rate (ESR), C-reactive protein (CRP), Human leukocyte antigen (HLA)-B27, GK), measurements for disease activity, functional status, spinal mobility, global assessment and health-related QoL.

### Disease specific instruments

The Bath Ankylosing Disease Activity Index (BASDAI) was used to evaluate disease activity [24]. The self-administered instrument consists of six questions, dealing with fatigue, spinal pain, joint pain and swelling, areas of localized tenderness and the severity and duration of morning stiffness. Functional status was assessed by Bath Ankylosing Spondylitis Functional Index (BASFI) [25, 26], which consists of 10 scales: eight visual analogue scales dealing with physical function and two scales reflecting the patient’s ability to deal with daily activities. Meanwhile, physical examinations were performed by orthopaedic residents to determine the patient’s physical mobility, including cervical rotation, tragus-to-wall distance, forward flexion (modified Schober index), lateral lumbar flexion, intermalleolar distance and the five physical parameters constitute the Bath Ankylosing Spondylitis Metrology Index (BASMI) score [27]. Bath Ankylosing Spondylitis Global Score (BAS-G) is comprised of two items questioning the effect of the disease on the patients’ overall well-being over the previous week and during the 6 months, respectively. The BASDAI, BASFI, BASMI and BAS-G scores have a final range from 0 to 10 and the higher scores of each parameter indicate higher disease activity, worse functional status, worse physical mobility and worse patients’ global assessment, respectively [28]. The ODI is a self-administered questionnaire measuring “back-specific function” on a 10 item scale with 6 response categories each. The 10 items include pain intensity, personal care, lifting, walking, sitting, standing, sleeping, sex life, social life, and traveling. Each item scores from 0 to 5. The total score of the ODI is calculated by adding all scores of applicable items, dividing the obtained score with the maximal total score and by multiplying the result for 100% to obtain a percentage score [19]. The total score of the index ranges from 0 to 100% and a higher score indicates more severe disability. Overall pain, fatigue and the severity and duration of morning stiffness were assessed by Visual Analogue Scale (VAS). All VAS were 10-cm horizontal lines.

### Generic instrument

Health-related QoL was evaluated by SF-36 consisting of 36 items, a generic instrument measuring eight different aspects of health status. The items of the SF-36 are grouped into eight subscales: physical functioning (10 items), role limitation due to physical health problems (4 items), bodily pain (2 items), general health perception (5 items), vitality (4 items), social functioning (2 items), role limitation due to emotional problems (3 items) and mental health (5 items). The first four subscales are combined to produce one summary measure: a Physical Component Summary (PCS) and the second four subscales are combined to produce another summary measure: a Mental Component Summary (MCS). These 35 questions are associated with the health status during the period of the last 4 weeks. The questionnaire also includes a single item that provides information about the assessment of the current health condition in comparison to the health condition a year ago. Items are scored in the range from 0 to 100 and a higher score means a better health status. The SF-36 takes about 5–10 min to complete [7, 8].

### Laboratory and radiographic measurements

Acute-phase reactants, including ESR and CRP levels were measured. ESR was measured by standard Westergren method (normal range 0-20 mm/h) and CRP by the immunoturbidimetric method (normal range 0-8 mg/L).

Long-cassette standing lateral radiographs of the spine were taken for measurements. GK was defined as the angle between the superior endplate of the maximally tilted upper end vertebra and the inferior endplate of the maximally tilted lower end vertebra [29]. Two groups were divided by GK: mild kyphotic group (GK < 70°,*n* = 176) and severe kyphotic group (GK ≥ 70°,*n* = 69) [30]. The difference of disease activity, functional status, spinal mobility and QoL between mild kyphotic and severe kyphotic groups was compared.

### Statistical analysis

The Spearman rank correlation test was applied to test correlations. Continuous variables were compared by Student’s t test. Hierarchal multiple regression analysis was used to investigate the contributions of demographic, clinical, laboratory and radiographic variables to physical function (PF) subscale of SF-36. To decrease the influence of other kind of parameters, we entered the variables in three successive conceptual blocks: (1) demographic variables, (2) laboratory and radiographic variables, and (3) clinical variables. Stepwise multiple regression analysis was then performed to identify the major contributors to PF subscale of SF-36. Collinearity diagnostics was applied to ensure that no multicollinearity existed in these two models. All statistical analyses were done using SPSS 19 (SPSS,USA). Descriptive data were presented as mean and standard deviation for continuous variables and as frequencies and percentages for categorical variables. A value of *P <* 0.05 was considered to be statistically significant.

## Results

### Patient characteristics

Two hundred and 45 patients (223 males and 22 females) with an average age

of 36.2 ± 10.9 years (range, 17–66 years) were enrolled in this study. Demographic, clinical, laboratory and radiographic characteristics of the study population are listed in Table 1. Mean age at the onset of symptoms was 24.7 ± 8.7 years (range, 8–57 years) and mean disease duration was 11.4 ± 8.0 years (range, 0–39 years). The ESR of 119 (49%) patients and the CRP of 176 (72%) patients were above normal range, respectively. Almost all the patients had a positive HLA-B27 allele (92%). Sixty-nine (28%) patients were severe kyphotic. Ninety-three (38%) patients had BASDAI ≥ 4. Mean BASFI and BASMI value were 3.2 (range 0–10) and 4.6 (range 0–10), respectively.

**Table 1:** Demographic, clinical, laboratory and radiographic characteristics of enrolled AS patients

Characteristics	Mean (SD) or frequencies (percentages)	Range
Age (years)	36.2 (10.9)	17–66
Male patients, n (%)	223 (91)	
Age at onset (years)	24.7 (8.7)	8–57
Disease duration (years)	11.4 (8.0)	0–39
Overall pain VAS (0-10 cm)	3.8 (2.3)	0–10
Fatigue VAS (0-10 cm)	4.1 (2.4)	0–10
Morning stiffness (degree) VAS (0-10 cm)	3.8 (2.9)	0–10
Morning stiffness (duration) (0-120 min)	36.7 (32.9)	0–120
ODI (%) (0–100)	31.8 (19.7)	0–94
Peripheral arthritis, n (%)	109 (44)	
NSAIDs intake, n (%)	156 (64)	
Anti-TNF drugs intake, n (%)	19 (8)	
ESR (mm/h)	27.0 (19.1)	2–126
CRP (mg/L)	24.7 (20.6)	0.2–128.4
HLA-B27+, n (%)	226 (92)	
GK (degrees)	61.0 (18.7)	22–122
BASDAI (0–10)	3.5 (2.0)	0–9
BASFI (0–10)	3.2 (2.6)	0–10
BASMI (0–10)	4.6 (3.1)	0–10
BAS-G (0–10)	5.0 (2.6)	0–10

*VAS* Visual Analogue Scale, *ODI* Oswestry Disability Index, *NSAIDs* Nonsteroidal Antiinflammatory Drugs, *TNF* tumor necrosis factor, *ESR* erythrocyte sedimentation rate, *CRP* C-reactive protein, *HLA-B27* Human leukocyte antigen-B27, *GK* global kyphosis, *BASDAI* Bath ankylosing spondylitis disease activity index, *BASFI* Bath ankylosing spondylitis functional index, *BASMI* Bath Ankylosing Spondylitis Metrology Index, *BAS-G* Bath Ankylosing Spondylitis Global Score

Mean scores for SF-36 subscales are shown in Table 2. Most affected subscales of SF-36 in AS patients were role physical (30.00 ± 39.02) and general health (35.19 ± 21.40). The physical component summary scale of SF-36 was significantly more impaired than the mental component summary scale (44.21 ± 20.79 vs. 54.19 ± 20.47; *P* < 0.001).

**Table 2:** Summary of each SF-36 subscale, physical component and mental component summary scale

	Mean (SD)	Range
Physical function (PF)	60.36 (25.01)	0–100
Role physical (RP)	30.00 (39.02)	0–100
Bodily pain (BP)	51.22 (22.07)	0–100
General health (GH)	35.19 (21.40)	0–100
Vitality (VT)	54.96 (17.78)	0–100
Social function (SF)	58.19 (23.11)	0–100
Role emotional (RE)	41.09 (43.93)	0–100
Mental health (MH)	62.52 (18.83)	12–100
Physical component summary (PCS)	44.21 (20.79)	1.25–95
Mental component summary (MCS)	54.19 (20.47)	7.25–97.50

### Relationship between BASDAI, BASFI, BASMI, ODI and SF-36 subscales

The scores of BASDAI, BASFI, BASMI and ODI had significant negative correlations with all SF-36 subscales. BASDAI had the strongest correlation with bodily pain (*r* = −0.616, *P* < 0.001). BASFI, BASMI and ODI were most correlated with PF subscale (*r* = −0.669, −0.523, and −0.725, respectively, *P* < 0.001) (Table 3).

**Table 3:** Correlations between BASDAI, BASFI, BASMI, ODI and SF-36 subscales

	BASDAI		BASFI		BASMI		ODI	
	*r*	*P*	*r*	*P*	*r*	*p*	*r*	*p*
Physical function	−0.394	<0.001	−0.669	<0.001	−0.523	<0.001	−0.725	<0.001
Role physical	−0.345	<0.001	−0.471	<0.001	−0.277	<0.001	−0.447	<0.001
Bodily pain	−0.616	<0.001	−0.554	<0.001	−0.298	<0.001	−0.689	<0.001
General health	−0.274	<0.001	−0.318	<0.001	−0.224	<0.001	−0.443	<0.001
Vitality	−0.380	<0.001	−0.396	<0.001	−0.227	<0.001	−0.388	<0.001
Social function	−0.292	<0.001	−0.466	<0.001	−0.296	<0.001	−0.554	<0.001
Role emotional	−0.315	<0.001	−0.328	<0.001	−0.169	0.008	−0.328	<0.001
Mental health	−0.319	<0.001	−0.288	<0.001	−0.173	0.007	−0.378	<0.001

*BASDAI* Bath ankylosing spondylitis disease activity index, *BASFI* Bath ankylosing spondylitis functional index, *BASMI* Bath Ankylosing Spondylitis Metrology Index, *ODI* Oswestry Disability Index

### Comparison of BASDAI, BASFI, BASMI and SF-36 subscales between mild Kyphotic group and severe Kyphotic group

**Fig. 1:**
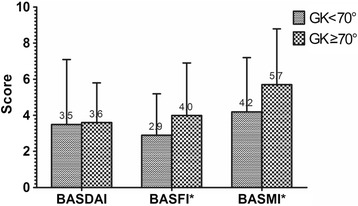
Comparison of BASDAI, BASFI and BASMI between mild kyphotic group (GK < 70°) and severe kyphotic group (GK ≥ 70°). BASFI and BASMI were significantly different between groups. *: *P* < 0.05

Statistically, no significant difference was found for BASDAI between mild kyphotic and severe kyphotic groups (*P* = 0.726). BASFI and BASMI scores of severe kyphotic group were significantly higher than those of mild kyphotic group, respectively (*P* = 0.005 and *P* = 0.001, respectively) (Fig. 1).

**Fig. 2:**
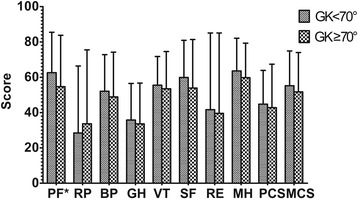
Comparison of SF-36 subscales between mild kyphotic group (GK < 70°) and severe kyphotic group (GK ≥ 70°). Only PF was significanly different between groups. *: *P* < 0.05

The quality of life of severe kyphotic group was worse than that of mild kyphotic group. The score of PF subscale in severe kyphotic group was significantly lower than those in mild kyphotic group (*P* < 0.05). There was no significant difference in other subscales of SF-36, PCS and MCS between the two groups (Fig. 2).

### Contributors and non-contributors to physical function (PF)

The independent variables including demographic, clinical, laboratory and radiographic variables were entered into the analysis in the following models to investigate the factors contributing variance to PF.

Results of hierarchic multiple regression analysis of the factors in relation to PF are shown in Table 4. Model 1 (demographic variables model) describes the contribution of demographic variables to PF. These variables contributed an overall *R* ^2^ of 6.8% (*P* < 0.05).Model 2 (laboratory and radiographic variables model)evaluates the contributions of laboratory and radiographic variables to PF. These variables increased 5.5% in the *R* ^2^ value (*R* ^2^ = 12.3%). Only CRP contributed significantly to PF in this model (*P* < 0.05). Model 3 (clinical variables model) shows the standard multiple regression analysis and all of the investigated variables were entered into the model to assess their contributions to PF. ODI (*P* < 0.001) and BASFI (*P* < 0.001) were significant determinants of PF. An additional 50.4% of the variance in PF was added by the clinical variables. The whole model could explain 62.7% of the total variance in PF.

**Table 4:** Hierarchic multiple regression analysis of demographic, clinical, laboratory and radiographic variables in relation to physical function (PF)

	Model 1		Model 2		Model 3	
	Standardized coefficient *P*		Standardized coefficient *P*		Standardized coefficient *P*	
Age	−0.104	0.191	−0.129	0.105	−0.066	0.231
Gender	−0.134	0.033	−0.097	0.123	−0.030	0.483
Disease duration	−0.135	0.087	−0.136	0.084	0.029	0.581
ESR			−0.005	0.944	−0.023	0.633
CRP			−0.215	0.003	0.019	0.698
GK			−0.118	0.071	0.002	0.958
Overall pain (VAS)					−0.040	0.486
Morning stiffness (degree) (VAS)					0.084	0.306
Morning stiffness (duration) (min)					−0.086	0.192
Fatigue (VAS)					−0.053	0.442
ODI					−0.432	<0.001
BASDAI					0.123	0.224
BASFI					−0.366	<0.001
BASMI					−0.106	0.054
BASG					−0.004	0.953
*R* ^*2*^ (%)	6.8		12.3		62.7	
Adjusted *R* ^*2*^ (%)	5.6		10.1		60.2	
F	24.297		23.717		15.778	

*ESR* erythrocyte sedimentation rate, *CRP* C-reactive protein, *GK* global kyphosis, *VAS* Visual Analogue Scale, *ODI* Oswestry Disability Index, *BASDAI* Bath ankylosing spondylitis disease activity index, *BASFI* Bath ankylosing spondylitis functional index, *BASMI* Bath Ankylosing Spondylitis Metrology Index, *BAS-G* Bath Ankylosing Spondylitis Global Score

*Model 1* demographic variables model, *Model 2* laboratory and radiographic variables model, *Model 3* clinical variables model

### Major contributors to physical function (PF)

The results of multiple stepwise regression analysis in PF are shown in Table 5. Higher ODI (B = −0.548, *P* < 0.001), BASFI (B = −3.303, *P* < 0.001) and BASMI (B = −0.969, *P* < 0.001) score were significantly associated with lower PF score. These variables could explain 61.3% of variance in PF. The analysis indicated that ODI (standardized coefficient = −0.431) contributed most in predicting PF statistically.

**Table 5:** Stepwise multiple regression analysis with physical function (PF) subscale as dependent variable

Independent variables	*B*	Standardized coefficient	*t*	*P*	*R* ^*2*^ (%)
Overall model					61.3
ODI	−0.548	−0.431	−7.641	<0.001	
BASFI	−3.303	−0.337	−5.617	<0.001	
BASMI	−0.969	−0.119	−2.393	0.017	

*ODI* Oswestry Disability Index, *BASFI* Bath ankylosing spondylitis functional index, *BASMI* Bath Ankylosing Spondylitis Metrology Index

## Discussion

Ankylosing spondylitis (AS) is characterized by inflammatory back pain and bony fusion of the axial skeleton [1]. It may lead to irreversible structural damage and the consequent reduction in functional status, limitation of physical mobility and poor QoL. The present study aimed to investigate QoL and correlation with disease activity, functional status, spinal mobility in AS patients.

In the current study, around 75% of the AS patients developed the first symptoms at an age which was younger than 30 years and less than 3% (around 2.4%) of patients had the onset age which was older than 45 years, which is similar to previous studies [1, 31]. In consideration of the above mentioned findings, AS is likely to develop in the early adulthood and affects patients in their most productive age which has posed a long-standing burden on both the individual and society, thus requiring a close attention. The peripheral joint involvement of AS in the previous studies is present in 20–51% of patients [16, 32]. In the current study, 45% of the patients had peripheral arthritis at a similar rate to the literature, and hips were the most commonly affected joints, confirming previous studies [33, 34].

With regard to QoL, our results were in line with previous studies by Ozgul et al. [3] and Vesović-Potić et al. [13], showing that role physical was the most affected subscale of SF-36, whereas mental health and physical function were least affected in AS patients, which was similar to the results of previous studies [12]. Some published literatures also indicated that AS affected mostly physical subscales of SF-36 [13, 14, 18, 35], which was supported by our findings: physical component summary (PCS) was significantly more impaired than mental component summary (MCS).

BASDAI, BASFI and BASMI with proven reliability and validity are commonly and widely used in the clinical assessment of AS patients [24, 25, 27]. In the present study, BASDAI and BASFI scores were significantly and negatively correlated with all SF-36 subscales. Likewise, Özdemir [18] reported that BASDAI and BASFI had significant negative correlations with all SF-36 subscales except for general health. On the contrary, some researchers [15] found BASDAI was only correlated with general health significantly and BASFI had a significant correlation only with emotional role. In the current study, BASDAI was most correlated with bodily pain, which is in line with the published results by Bodur et al. [36]. Similar to previous studies [18, 36], BASFI had the strongest correlation with physical function subscale of SF-36. The current study indicated that BASMI was also correlated with all of the subscales of SF-36, which is in accordance with the results reported by Bodur et al. [36]. However, a few studies [13, 17] reported that only two subscales (physical functioning and general health) of SF-36 were negatively correlated with BASMI index, which may be owing to the relatively small sample size of these studies. Considering these findings, the disease-specific instruments (BASDAI, BASFI, BASMI) for AS correlated well with the generic instrument (SF-36) and it could be concluded that the broad scope of SF-36 might reflect the health status in AS populations adequately and accurately.

In advanced stage of AS, patients often develop thoracolumbar or cervical kyphosis. The GK is a radiographic parameter which is used to evaluate the severity of kyphosis in sagittal plane [29, 30, 37]. Decreased volume of thoracic and abdominal cavity caused by the increased GK will cause the compression of important organs (heart, lung, intestine, etc) and further lead to cardiopulmonary and gastrointestinal dysfunction. In addition, the increased GK will lead to enhanced muscle tension of the back and severe fatigue, thus impairing the QoL. Shin et al. [20] investigated the correlation between clinical outcome and spinopelvic parameters in AS, but they did not evaluated the relationship between GK and clinical variables. Moreover, few studies reported the relationship between GK and QoL evaluated by SF-36 in AS patients. Our study showed that AS patients in severe kyphotic group had significantly higher BASFI and BASMI scores than those in mild kyphotic group. Compared to mild kyphotic group, patients in severe kyphotic group had significantly lower score in physical function subscale of SF-36. These results suggested that development of kyphotic deformity might contribute to impairment of physical function and spinal mobility. Therefore, the prevention of GK progression in early stage and correction of GK in advanced stage are crucial for the treatment of AS. However, no significant difference was observed for BASDAI between these two groups, the possible explanation might be that the degree of disease activity was not always consistent with the severity of kyphosis in AS, which meant that an AS patient with severe kyphosis did not necessarily have high disease activity.

PF subscale of SF-36 describes the limitations in performing physical activities due to the deterioration of physical health [38] and it has been identified as a main outcome domain in AS [26, 39]. Limitations in physical function may influence the ability to remain in employment and to participate in leisure and domestic activities. Also, the ability to fulfill social roles in family and society may thereby be restricted, thus influencing the person’s emotional state [11]. Therefore, the whole well-being of patients would be disrupted. To date, no studies have weighed the relative impact of demographic, radiographic, laboratory and clinical variables on PF in a hierarchical regression model in AS patients. In the current study, it was noted that each model made significant contributions to PF (*P* < 0.05). In demographic model, gender contributed significantly to PF (*P* = 0.033). The standardized coefficient was negative which meant female patients experienced lower PF score than males. Previous studies investigating gender-attributable differences with respect to QoL in AS have also shown that female gender was associated with lower QoL [40–43]. However, they mainly showed lower QoL in females regarding mental health, rather than physical health [40]. In laboratory and radiographic model, only CRP contributed significantly to PF (standardized coefficient = −0.215, *P* = 0.003) which suggested that increased CRP level was correlated with decreased PF. Similarly, it was revealed by Cansu et al. [44] that elevated CRP was associated with poor functional status. These findings indicated that the reduction of CRP level was helpful to the improvement of physical function in AS patients. Clinical variables made the most contribution to PF after correction for other factors, adding an additional 50.4% of the variance in PF. Multiple stepwise regression analysis was also applied to investigate major contributors to PF in AS patients, showing that BASFI and BASMI were the independently associated factors with PF. Similarly, Vesović-Potić et al. [13] developed a regression model to identify variables associated with physical function subscale of SF-36 and reported that BASFI score was the independently associated factor. Besides, Ariza-Ariza et al. [14] reported that spinal mobility (evaluated by BASMI) was the main factor associated with PF in AS patients. With proven reliability and validity, ODI has become one of the most commonly used measures of disability in back pain [45–48]. However, few studies have investigated the relationship between the pain-related disability measured by ODI and QoL in AS patients. In the present study, significant negative correlations were observed between ODI and all SF-36 subscales. Notably, ODI was also a major contributor to PF in the multiple stepwise regression model. It seems reasonable because both ODI and PF evaluate the problems associated with physical activities in daily life. In consideration of these findings, effective treatment of low back pain and valid management strategies to slow the progressive loss of spinal mobility were beneficial to the improvement of QoL. In the current study, BASDAI was not a major predictor of PF. However, in contrast to our study, Fernández-Carballido et al. [49] suggested that disease activity (measured by BASDAI) was a major determinant of QoL and physical function in patients with axial spondyloarthritis (including AS). One possible explanation of the distinct results might be that the patients enrolled in their study were in early stage of the disorder, during which period physical function was significantly influenced by pain, fatigue and stiffness just evaluated by BASDAI.

The limitations of the present study should be acknowledged. Firstly, the current study is a retrospective design. Secondly, with our department specializing in spine deformity correction, much more kyphotic patients who needed surgery came to our center. It is possible that the proportion of advanced AS patients is high in this study, leading to research bias (e.g. worse clinical and radiographic outcomes). Further prospective longitudinal studies are required to establish the definitive relationship between various clinical outcomes and QoL, and identify the correlation between radiographic parameters and QoL in AS patients from different populations.

## Conclusions

The present study demonstrated that impaired QoL is significantly correlated with high disease activity, poor functional status and decreased spinal mobility in AS patients. GK may be an important radiographic parameter that affects functional status, spinal mobility and QoL in AS patients. ODI, BASFI, BASMI are the major contributors to PF subscale of SF-36. Understanding these complex relationships in AS patients is of utmost importance for both physicians and surgeons to facilitate counseling and management of AS.

## Abbreviations

AS: Ankylosing spondylitis
BASDAI: Bath Ankylosing Spondylitis Disease Activity Index
BASFI: Bath Ankylosing Spondylitis Functional Index
BAS-G: Bath Ankylosing Spondylitis Global score
BASMI: Bath Ankylosing Spondylitis Metrology Index
CRP: C-reactive protein
ESR: Erythrocyte sedimentation rate
GK: Global kyphosis
HLA-B27: Human leukocyte antigen-B27
MCS: Mental Component Summary
NSAIDs: Nonsteroidal antiinflammatory drugs
ODI: Oswestry Disability Index
PCS: Physical Component Summary
QoL: Quality of life
SF-36: Short Form-36
TNF: Tumor necrosis factor
VAS: Visual analogue scale

## References

[1] Braun J, Sieper J (2007). Ankylosing spondylitis. Lancet.

[2] Ward MM (1998). Quality of life in patients with ankylosing spondylitis. Rheum Dis Clin N Am.

[3] Ozgul A, Peker F, Taskaynatan MA, Tan AK, Dinçer K, Kalyon TA (2006). Effect of ankylosing spondylitis on health-related quality of life and different aspects of social life in young patients. Clin Rheumatol.

[4] Fitzpatrick R (1993). The measurement of health status and quality of life in rheumatologic disorders. Baillieres Clin Rheumatol.

[5] Ortiz Z, Shea B, Garcia Dieguez M, Boers M, Tugwell P, Boonen A, Wells G (1999). The responsiveness of generic quality of life instruments in rheumatic diseases. A systematic review of randomized controlled trials. J Rheumatol.

[6] Ward MM (1999). Health-related quality of life in ankylosing spondylitis: a survey of 175 patients. Arthritis Care Res.

[7] Gyatt GH, Feeney DH, Patric DL (1993). Measuring health-related quality of life. Ann Intern Med.

[8] Brazier JE, Harper R, Jones NM, O'Cathain A, Thomas KJ, Usherwood T, Westlake L (1992). Validating the SF-36 health survey questionnaire: new outcome measure for primary care. BMJ.

[9] Ware JE (2000). Using generic measures of functional health and well-being to increase understanding of disease burden. Spine.

[10] Chorus AM, Miedema HS, Boonen A, Van Der Linden S (2003). Quality of life and work in patients with rheumatoid arthritis and ankylosing spondylitis of working age. Ann Rheum Dis.

[11] Dagfinrud H, Mengshoel AM, Hagen KB, Loge JH, Kvien TK (2004). Health status of patients with ankylosing spondylitis: a comparison with the general population. Ann Rheum Dis.

[12] Jajić Z, Rajnpreht I, Kovačić N, Lukić IK, Velagić V, Grubišić F, Marušić A, Grčević D (2012). Which clinical variables have the most significant correlation with quality of life evaluated by SF-36 survey in Croatian cohort of patient with ankylosing spondylitis and psoriatic arthritis. Rheumatol Int.

[13] Vesović-Potić V, Mustur D, Stanisavljević D, Ille T, Ille M (2009). Relationship between spinal mobility measures and quality of life in patients with ankylosing spondylitis. Rheumatol Int.

[14] Ariza-Ariza R, Hernández-Cruz B, Navarro-Sarabia F (2003). Physical function and health-related quality of life of Spanish patients with ankylosing spondylitis. Arthritis Rheum.

[15] Turan Y, Duruöz MT, Cerrahoglu L (2007). Quality of life in patients with ankylosing spondylitis: a pilot study. Rheumatol Int.

[16] Bostan EE, Borman P, Bodur H (2003). Functional disability and quality of life in patients with ankylosing spondylitis. Rheumatol Int.

[17] Hamdi W, Azzouz D, Saadellaoui K, Daoud L, Kochbati S, Ben Hamida A, Zouari B, Kochir MM (2007). Correlations between bath Ankylosing Spondylitis metrology index (BASMI), functional, structural, enthesopathy, disease activity index, and quality of life in 120 patients with ankylosing spondylitis. Ann Rheum Dis.

[18] Özdemir O (2011). Quality of life in patients with ankylosing spondylitis: relationships with spinal mobility, disease activity and functional status. Rheumatol Int.

[19] Fairbank JC, Couper J, Davies JB, O’Brien JP (1980). The Oswestry low back pain questionnaire. Physiotherapy.

[20] Shin JK, Lee JS, Goh TS, Son SM (2014). Correlation between clinical outcome and spinopelvic parameters in ankylosing spondylitis. Eur Spine J.

[21] Arun R, Dabke HV, Mehdian H (2011). Comparison of three types of lumbar osteotomy for ankylosing spondylitis: a case series and evolution of a safe technique for instrumented reduction. Eur Spine J.

[22] Zhang HQ, Huang J, Guo CF, Liu SH, Tang MX (2014). Two-level pedicle subtraction osteotomy for severe thoracolumbar kyphotic deformity in ankylosing spondylitis. Eur Spine J.

[23] Van der Linden S, Valkenburg HA, Cats A (1984). Evaluation of diagnostic criteria for ankylosing spondylitis: a proposal for modification of the New York criteria. Arthritis Rheum.

[24] Garrett S, Jenkinson T, Kennedy LG, Whitelock H, Gaisford P, Calin A (1994). A new approach to defining disease status in AS: the bath Ankylosing Spondylitis disease activity index. J Rheumatol.

[25] Calin A, Garrett S, Whitelock H, Kennedy LG, O'Hea J, Mallorie P, Jenkinson T (1994). A new approach to defining functional ability in ankylosing spondylitis: the development of the bath Ankylosing Spondylitis functional index (BASFI). J Rheumatol.

[26] Ruof J, Sangha O, Stucki G (1999). Comparative responsiveness of 3 functional indices in ankylosing spondylitis. J Rheumatol.

[27] Jenkinson TR, Mallorie PA, Whitelock HC, Kennedy LG, Garrett SL, Calin A (1994). Defining spinal mobility in ankylosing spondylitis (AS): the bath AS metrology index(BASMI). J Rheumatol.

[28] Chen CH, Chen HA, Liao HT, Liu CH, Tsai CY, Chou CT (2015). The clinical usefulness of ESR, CRP, and disease duration in ankylosing spondylitis: the product of these acute-phase reactants and disease duration is associated with patient’s poor physical mobility. Rheumatol Int.

[29] Qian BP, Qiu Y, Wang B, Sun X, Zhu ZZ, Jiang J, Ji ML (2012). Pedicle subtraction osteotomy through pseudarthrosis to correct thoracolumbar kyphotic deformity in advanced ankylosing spondylitis. Eur Spine J.

[30] Qian BP, Wang XQ, Qiu Y, Jiang H, Ji ML, Jiang J (2013). An exon polymorphism of programmed cell death 1 gene is associated with both the susceptibility and thoracolumbar kyphosis severity of ankylosing spondylitis in a Chinese Han population. J Orthop Sci.

[31] Feldtkeller E, Khan MA, van der Heijde D, van der Linden S, Braun J (2003). Age at disease onset and diagnosis delay in HLA-B27 negative vs. positive patients with ankylosing spondylitis. Rheumatol Int.

[32] Ward MM, Kuzis S (1999). Validity and sensitivity to change of spondylitis-specific measures of functional disability. J Rheumatol.

[33] Dalyan M, Güner A, Tuncer S (1999). Disability in ankylosing spondylitis. Disabil Rehabil.

[34] Van der Linden S, Van der Heijde D, Ruddy S, Harris ED, Sledge CB (2001). Ankylosing spondylitis. Kelley’s textbook of rheumatology.

[35] Exarchou S, Lie E, Lindström U, Askling J, Forsblad-d'Elia H, Turesson C, Kristensen LE, Jacobsson LT (2016). Mortality in ankylosing spondylitis: results from a nationwide population-based study. Ann Rheum Dis.

[36] Bodur H, Ataman S, Rezvani A (2011). Quality of life and related variables in patients with ankylosing spondylitis. Qual Life Res.

[37] Fu J, Wu M, Liang Y, Song K, Ni M, Zhang Y, Chen J (2016). Differences in cardiovascular manifestations between ankylosing spondylitis patients with and without kyphosis. Clin Rheumatol.

[38] Ware JE (2000). SF-36 health survey update. Spine.

[39] Van der Heijde DMFM, Bellamy N, Calin A, Dougados M, Khan MA, van der Linden S (1997). Preliminary core sets for endpoints in ankylosing spondylitis. Assessments in Ankylosing Spondylitis working group. J Rheumatol.

[40] Webers C, Essers I, Ramiro S, Stolwijk C, Landewé R, van der Heijde D (2016). Gender-attributable differences in outcome of ankylosing spondylitis: long-term results from the outcome in Ankylosing Spondylitis international study. Rheumatology (Oxford).

[41] Tournadre A, Pereira B, Lhoste A, Dubost JJ, Ristori JM, Claudepierre P, Dougados M, Soubrier M (2013). Differences between women and men with recent-onset axial spondyloarthritis: results from a prospective multicenter French cohort. Arthritis Care Res.

[42] de Carvalho HM, Bortoluzzo AB, Goncalves CR (2012). Gender characterization in a large series of Brazilian patients with spondyloarthritis. Clin Rheumatol.

[43] Dagfinrud H, Kjeken I, Mowinckel P, Hagen KB, Kvien TK (2005). Impact of functional impairment in ankylosing spondylitis: impairment, activity limitation, and participation restrictions. J Rheumatol.

[44] Cansu DU, Calışır C, Savaş Yavaş U, Kaşifoğlu T, Korkmaz C (2011). Predictors of radiographic severity and functional disability in Turkish patients with ankylosing spondylitis. Clin Rheumatol.

[45] Smeets R, Köke A, Lin CW, Ferreira M, Demoulin C (2011). Measures of function in low back pain/disorders: low back pain rating scale (LBPRS), Oswestry disability index (ODI), progressive Isoinertial lifting evaluation (PILE), Quebec back pain disability scale (QBPDS), and Roland-Morris disability questionnaire (RDQ). Arthritis Care Res.

[46] Fairbank JC, Pynsent PB (2000). The Oswestry disability index. Spine (Phila Pa 1976).

[47] Roland M, Fairbank J (2000). The Roland-Morris disability questionnaire and the Oswestry disability questionnaire. Spine (Phila Pa 1976).

[48] Davidson M, Keating JL (2002). A comparison of five low back disability questionnaires: reliability and responsiveness. Phys Ther.

[49] Fernández-Carballido C, Navarro-Compán V, Castillo-Gallego C (2017). Disease activity as a major determinant of quality of life and physical function in patients with early axial spondyloarthritis. Arthritis Care Res (Hoboken).

